# Author's Reply to “Digital Devices for Arrhythmia Detection: What Is Still Missing?”

**DOI:** 10.1002/clc.70092

**Published:** 2025-02-06

**Authors:** Martin Manninger, David Zweiker, Tatevik Hovakimyan, Paweł T. Matusik, Sergio Conti, Pierre Ollitrault, Aapo Aro, Bart A. Mulder, Wolfgang Dichtl, Christian‐Hendrik Heeger, Rachel M. A. ter Bekke, Enes Elvin Gul, Bob Weijs, Ann‐Kathrin Rahm, Angeliki Darma, Banu Evranos, Avi Sabbag, Kgomotso Moroka, Vassil Traykov, Jacob Moesgaard Larsen, Gisella Rita Amoroso, Stijn Evens, William F. McIntyre, Dominik Linz

**Affiliations:** ^1^ Department of Internal Medicine Division of Cardiology Medical University of Graz Graz Austria; ^2^ Department of Cardiology Maastricht University Medical Centre, Cardiovascular Research Institute Maastricht (CARIM) Maastricht the Netherlands; ^3^ Department of Cardiology and Intensive Care Clinic Ottakring Vienna Austria; ^4^ Department of Cardiac Arrhythmology Nork‐Marash Medical Center Yerevan Armenia; ^5^ Department of Electrocardiology Institute of Cardiology, Jagiellonian University Medical College, Faculty of Medicine Kraków Poland; ^6^ Department of Electrocardiology St. John Paul II Hospital Kraków Poland; ^7^ Department of Cardiac Electrophysiology ARNAS Civico Hospital Palermo Italy; ^8^ Department of Cardiology Electrophysiology Unit, Caen University Hospital Caen France; ^9^ Heart and Lung Center, Division of Cardiology, University of Helsinki and Helsinki University Hospital Helsinki Finland; ^10^ Department of Cardiology University Medical Centre Groningen University of Groningen Groningen the Netherlands; ^11^ Department of Internal Medicine III Medical University of Innsbruck Innsbruck Austria; ^12^ Department of Rhythmology University Heart Center Lübeck, University Hospital Schleswig‐Holstein Lübeck Germany; ^13^ German Center for Cardiovascular Research (DZHK), Partner Site Lübeck Germany; ^14^ Department of Rhythmology Asklepios Klinik Hamburg Altona Hamburg Germany; ^15^ Division of Cardiac Electrophysiology, Madinah Cardiac Center Madinah Saudi Arabia; ^16^ Medicine Hospital Istanbul Atlas University Istanbul Turkey; ^17^ Zuyderland Medical Centre Heerlen & Maastricht UMC Heerlen the Netherlands; ^18^ Department of Cardiology, Angiology and Pneumology Heidelberg University Hospital Heidelberg Germany; ^19^ Heidelberg Center for Heart Rhythm Disorders (HCR), Heidelberg University Hospital Heidelberg Germany; ^20^ German Center for Cardiovascular Research (DZHK), Partner Site Heidelberg/Mannheim Heidelberg Germany; ^21^ Department of Electrophysiology Heart Centre Leipzig Leipzig Germany; ^22^ Department of Cardiology Faculty of Medicine Hacettepe University Ankara Turkey; ^23^ Leviev Heart Center, Sheba Medical Center, Affiliated With the School of Medicine Tel Aviv University Tel Aviv Israel; ^24^ Department of Cardiology University of the Free State Bloemfontein South Africa; ^25^ Department of Invasive Electrophysiology and Cardiac Pacing Acibadem City Clinic Tokuda Hospital Sofia Bulgaria; ^26^ Department of Cardiology, Department of Clinical Medicine Aalborg University Hospital Aalborg University Aalborg Denmark; ^27^ Divisione di Cardiologia, Dipartimento Medico Specialistico, Ospedale “SS Annunziata” Savigliano Italy; ^28^ Qompium Hasselt Belgium; ^29^ Population Health Research Institute Hamilton Canada; ^30^ Department of Medicine McMaster University Hamilton Canada; ^31^ Department of Biomedical Sciences Faculty of Health and Medical Sciences University of Copenhagen Copenhagen Denmark

We thank Kataoka and Imamura for their interest in our recently published survey on physician's preferences in the use of novel digital devices in the management of patients with atrial fibrillation (AF) [1, 2].

Our survey shows, that digital devices are beginning to be implemented in clinical practice. We respectfully disagree with Kataoka and Imamura that the debate on the type of monitoring technology is not critical at the moment. Our international group of authors strongly believe that the switch to increased patient involvement, to patient‐initiated rhythm monitoring and to telemedical care require physician (and not industry) driven education on the technologies used, recommendations for specific diagnostic pathways, cost‐effectiveness analyses and outcome‐centered research [3, 4, 5, 6, 7, 8].

The clinical scenarios presented in this survey do not aim to give specific recommendations on diagnostic pathways but aim to reflect common clinical scenarios we experience as physicians in daily clinical practice. Diagnostic pathways for these scenarios are reflected in current clinical practice guidelines: There is a clear recommendation to confirm AF in symptomatic patients and to screen for AF in patients at risk [5, 6]. We agree with the Kataoka and Imamura that the duration of monitoring is crucial and still one of the unanswered questions, but first, there is evolving data in this field and second, screening duration using novel digital devices is often self‐determined by patients [9, 10].

We agree with Kataoka and Imamura's opinion that AF screening in the general population shows questionable benefit. As the Apple Heart Study showed, even the number needed to screen to diagnose AF is exceptionally high [11]. Consequently, the number needed to screen to show clinical benefit in this population are expected to be even higher. However, the presented patient scenarios reflect opportunistic testing for AF in patients with risk factors for adverse outcomes as a result of under‐detected AF, which represents a clinical challenge in several outpatient clinics [6].

We believe that novel digital devices for rhythm monitoring provide important diagnostic tools for screening, diagnosis and management of AF when used in the right populations at risk/patients. These devices may increase patient's adherence to treatment and even decrease anxiety related to known recurrences of benign arrhythmias. Referring to the author's question: “Digital Devices for Arrhythmia Detection: What is Still Missing?”: More physician and patient education on the potential of novel digital devices is required to achieve diagnostic pathways as suggested by the EHRA practical guide [5].
